# A Possible Role for Platelet-Activating Factor Receptor in Amyotrophic Lateral Sclerosis Treatment

**DOI:** 10.3389/fneur.2018.00039

**Published:** 2018-02-06

**Authors:** Marcelo R. S. Briones, Amanda M. Snyder, Renata C. Ferreira, Elizabeth B. Neely, James R. Connor, James R. Broach

**Affiliations:** ^1^Department of Health Informatics, Escola Paulista de Medicina, UNIFESP, São Paulo, São Paulo, Brazil; ^2^Department of Biochemistry, Penn State College of Medicine, Institute for Personalized Medicine, Hershey, PA, United States; ^3^Department of Neurosurgery, Penn State College of Medicine, Hershey, PA, United States; ^4^Department of Neurology and Neurosurgery, Escola Paulista de Medicina, UNIFESP, São Paulo, São Paulo, Brazil

**Keywords:** amyotrophic lateral sclerosis, platelet-activating factor, neuroinflammation, anti-inflammatory, cytokines

## Abstract

Amyotrophic lateral sclerosis (ALS) is the third most prevalent neurodegenerative disease affecting upper and lower motor neurons. An important pathway that may lead to motor neuron degeneration is neuroinflammation. Cerebrospinal Fluids of ALS patients have increased levels of the inflammatory cytokine IL-18. Because IL-18 is produced by dendritic cells stimulated by the platelet-activating factor (PAF), a major neuroinflammatory mediator, it is expected that PAF is involved in ALS. Here we show pilot experimental data on amplification of PAF receptor (PAFR) mRNA by RT-PCR. PAFR is overexpressed, as compared to age matched controls, in the spinal cords of transgenic ALS SOD1-G93A mice, suggesting PAF mediation. Although anti-inflammatory drugs have been tested for ALS before, no clinical trial has been conducted using PAFR specific inhibitors. Therefore, we hypothesize that administration of PAFR inhibitors, such as Ginkgolide B, PCA 4248 and WEB 2086, have potential to function as a novel therapy for ALS, particularly in SOD1 familial ALS forms. Because currently there are only two approved drugs with modest effectiveness for ALS therapy, a search for novel drugs and targets is essential.

## Introduction

Amyotrophic lateral sclerosis (ALS), a motor neuron disease, is the third most prevalent neurodegenerative disease (4 cases per 100,000 people), being Alzheimer’s disease and Parkinson’s disease the first and second, respectively (1). ALS affects upper and lower motor neurons with pronounced degeneration of Alpha motor neurons that innervate extrafusal fibers of skeletal muscle (2). The clinical manifestations of ALS are muscle atrophy, dysphagia, dysarthria, spasticity, hyperreflexia, fasciculation, and respiratory failure. Neuroimaging showed that corticospinal tract degeneration correlates with a rapid disease progression (3, 4). ALS is a progressive, irreversible and fatal neurodegenerative disease for which no effective therapy exists.

Previous observations have established that only 10% of ALS patients have a family history of the disease, which means that 90% of patients have no near relatives who have presented with the disease. Twin studies have estimated ALS heritability to be 60–70% (5), suggesting that many patients who present with sporadic ALS (SALS) may also have an underlying genetic cause. C9orf72 and SOD1 are considered the “major” ALS-causing genes. Mutations in C9orf72 are observed in 38% familial ALS (FALS) and 8% SALS while mutations in SOD1 have been reported in 13% FALS and about 1% SALS (6, 7). Since SOD1 has been the first ALS-causing gene described, in 1993, transgenic mouse models have been well established for the SOD1 ALS and have been used in several studies bearing on the basic mechanisms of ALS. SOD1 ALS is caused by genetic gain of function and the mouse model has the G93A substitution, the most widely used transgenic mouse lineage (8).

Motor neuron degeneration may be cause by a plethora of pathways. An important one is neuroinflammation. Lymphocyte permeation and microglia activation are present in post-mortem spinal cord samples (9–11). A study on 10 ALS patients using positron emission tomography showed microglial activation in the brain and that the intensity of activation correlates with the severity of clinical deficits (12). Astrocytes also play an important role in ALS pathogenesis. Rodent astrocytes expressing human mutated SOD1 kills motor neurons but not spinal GABAergic or dorsal root ganglion neurons (13). It has been shown that among all inflammation-related IL-1 family cytokines (IL-1β, IL-18, IL-33, IL-37) and their endogenous inhibitors (IL-1Ra, sIL-1R2, IL-18BP, sIL-1R4) only IL-18 and its endogenous inhibitor, IL-18BP, are significantly increased in CSF of patients with ALS as measured by the ELISA method (14). The increase of total free IL-18 suggests the activation of IL-18-cleaving inflammasome. Activated IL-18 was detected in brain of SALS patients by *in situ* immune-histochemistry (15). This may indicate the involvement of cytokines in ALS physiopathology. Whether IL-18 upregulation in ALS patients is a consequence of inflammation or one of the causes of the pathology still needs to be tested.

Currently, two medications are approved by the FDA to treat ALS: Riluzole and Edaravone. Riluzole is an antiglutamate agent, noncompetitive NMDA receptor antagonist, known to inactivate voltage-gated sodium channels and decrease repetitive firing of action potentials (16–18). The proposed mechanism of action is anti-excitotoxity (19). Two prospective, double-blind, placebo-controlled trials in ALS patients show that riluzole appears to slow the disease progression and it may improve survival in patients with bulbar onset (20–22). However, a review combining results of three clinical trials showed that it confers a modest improvement in survival although providing relief of respiratory symptoms and some benefit on both bulbar and limb function (23, 24).

A new drug, Edaravone, was approved in 2017 (25). This drug is a free radical scavenger approved in 2011 in Japan for disorders associated with acute ischemic stroke (26). The first efficacy and safety trial do not to demonstrate edaravone efficacy in a confirmatory study with primary outcome based on the ALS functional rating scale (ALSFRS-R) scores (27). A *post hoc* subgroup analysis of this first clinical trial identify a group of patients were edaravone exhibited efficacy (28). This group was defined as patients with diagnostic of definite or probable ALS according to El Escorial, disease onset within two years and greater-efficacy-expected subpopulation within the efficacy-expected population with% forced vital capacity of ≥80%, and ≥2 points for all item scores in the revised ALSFRS-R score before treatment (28). In another phase 3, randomized, double-blind, parallel-group study with this *post hoc* subgroup only a small subset of people showed a smaller decline of ALSFRS-R score compared with placebo suggesting that edaravone may not be effective in all ALS patients (29).

Over 50 different drugs were tested for ALS with the majority failing to demonstrate efficacy. Classification of compounds tested by pathophysiological category were antiapoptotic, anti-inflammatory, antiexcitotoxicitory, antioxidant, antiaggregation, neuroprotective, and neurotrophic growth factor (16). Because neuroinflammation is involved in ALS pathogenesis a variety of anti-inflammatory drugs were tested. However, most of them fail to slow disease progression. For example, minocycline had harmful effects, and recombinant human erythropoietin, glatiramer acetate and thalidomide had no impact in disease progression in randomized, double blind, placebo controlled trials (30–33). A recent phase IIA clinical trial using fingolimod (NCT01786174; www.clinicaltrials.gov), a sphingosine 1-phosphate receptor modulator approved for the treatment of relapsing-remitting multiple sclerosis, demonstrated that the circulating lymphocytes decreased with treatment with significant downregulation of immuno-related genes (34). Two ongoing clinical trial using Ibudilast, a non-selective phosphodiesterase 4 inhibitor, are evaluating both neuroinflammation, safety and tolerance (NCT02714036, NCT02238626; www.clinicaltrials.gov).

Proinflammatory mediators modulate neuroinflammation and are also targets for ALS therapy. A study using IL-6 receptor antagonist Tocolizumab showed a decrease in cytokines proinflammatory secretion and a phase two trial is ongoing (NCT02469896; www.clinicaltrials.gov) (35). A pilot studies with a IL-1 receptor antagonist Anakira do not showed significant reduction in disease progression with antibodies against the drug found between 24 and 52 weeks of treatment (NCT01277315; www.clinicaltrials.gov) (36). Masitinib, a tyrosine-kinase inhibitor, is capable of controlling microgliosis and significantly prolonged survival in a pre-clinical trial using SOD1 (G93A) rat model (37).

Several alterations in brain chemistry are associated with ALS ranging from glutamate imbalance in upper motor neuron synapses, inflammation and astrocyte activation. Despite its demonstrated role in other neurological disorders (38), platelet-activating factor (PAF), also known as PAF-acether or acetyl-glyceryl-ether-phosphorylcholine, is a very important mediator of inflammatory response. It is a potent phospholipid activator and mediator of several leukocyte functions, platelet aggregation and degranulation, inflammation, and anaphylaxis. It is also involved in changes to chemotaxis of leukocytes, vascular permeability, oxidative burst, and increased arachidonic acid metabolism in phagocytes. High PAF levels are associated with a variety of medical conditions including: allergic reactions, multiple sclerosis, stroke, myocardial infarction, colitis, inflammation of the large intestine, and sepsis. However, PAF has not been characterized in ALS.

Platelet-activating factor is produced by several cell types, especially those involved in host immunity, such as platelets, macrophages, neutrophils, monocytes, and endothelial cells. PAF is constitutively produced in low levels by these cells and its synthesis is controlled by PAF acetylhydrolases activity *via* remodeling (lyso-PC acetyltransferases, LPCAT 1 and 2) or by *de novo* synthesis *via* phosphocholinetransferase (PAF-PCT) (39). In response to specific stimuli it is produced in larger quantities by inflammatory cells (40). PAF acts on a specific receptor, PAFR, expressed in mature and immature dendritic cells (41). Because PAF induces monocyte-derived dendritic cells but not macrophages to secrete IL-12 and IL-18 (37), it is expected that PAF, and it receptor, are involved in ALS as the source of increase IL-18 in ALS patients might be PAF activated dendritic cells. Therefore, the upregulation of PAFR in ALS patients, or the ALS-SOD1 mouse experimental model, might be suggestive of a PAF role in ALS.

## Hypothesis

We hypothesize a possible role for PAF receptor inhibitors as a novel therapy for ALS, particularly in SOD1-familial forms.

## Pilot Study Results

Here, we present a pilot study data on upregulation of PAFR in the ALS experimental mouse model. All animal experiments were performed in accordance with protocol #46763 approved by the IACUC of Pennsylvania State University. Experimental mice (SOD1-G93A strain, Jackson Laboratories) and control mice (C57BL/6J, Jackson Laboratories) were sacrificed at 110 days of age (symptomatic). Experimental mice in the symptomatic group are not severely affected at that time point and do retain normal feeding and grooming behavior although locomotion is affected, as evaluated by the rotarod performance test. Animals were deeply anesthetized using KAX (100 mg/kg ketamine, 10 mg/kg xylazine, and 3 mg/kg acepromazine, to be injected i.p. at a weight-adjusted dose of 0.1 mL/10 g bodyweight). The level of anesthesia was assessed by lack of response to toe and tail pinch, followed by cardiac puncture to remove blood and then exsanguination. Mice were decapitated immediately following exsanguination with sharpened scissors. Spinal cord tissue was removed and frozen in liquid nitrogen.

**Figure 1:**
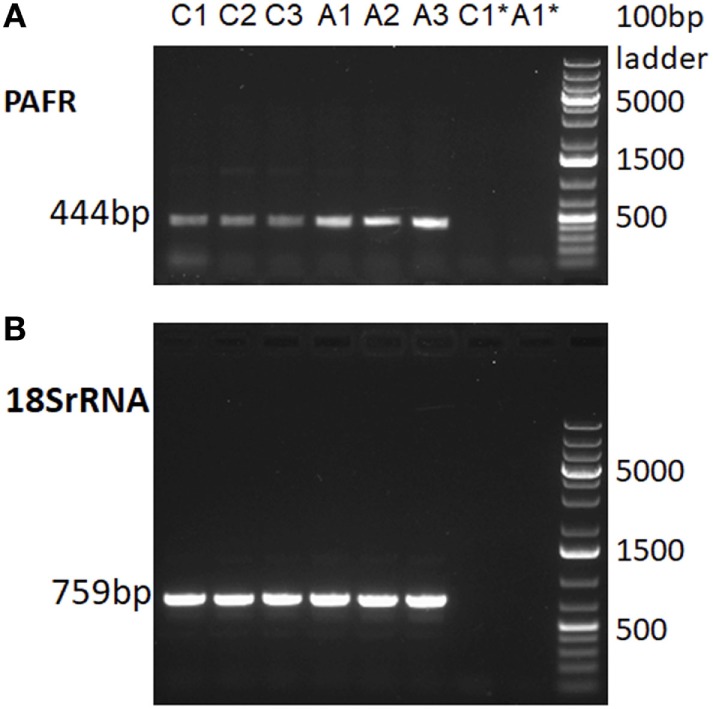
RT-PCR of platelet-activating factor receptor (PAFR) mRNA. In **(A)**, amplification with PAFR specific primers in ALS/SOD1-G93A mice (A1, A2, and A3) and in BL6 controls (C1, C2, and C3). In **(B)**, amplification of 18S rRNA for RNA load control in the same samples as in (A). PAFR yields a 444 bp amplicon and 18S rRNA a 759 bp amplicon. Primer annealing temperatures are 62°C for PAFR and 63°C for 18SrNA. C1* and A1* indicate RT-PCR controls without reverse transcriptase to show that amplicons are entirely dependent on reverse transcriptase activity and therefore not due to DNA contamination in RNA preparations.

Lumbar sections of the spinal cord were dissected and RNA isolated using the DNA/RNA extraction kit (DNAEssy, Qiagen) and quantitated using Nanodrop (ThermoFisher Scientific). Three ALS mice and three age matched controls were used in RT-PCR with SuperScript IV reverse transcriptase and PCR kit. PCR was carried with PAFR specific primers (87F-5′-GGTGACTTGGCAGTGCTTTG and 530R-5′-CACGTTGCACAGGAAGTTGG) located in two different exons (positions 87 in exon 1 and position 15,510 in exon 2 of PAFR gene). For RNA load control amplification of 18S rRNA was used (primers 757F-5′- CCCCTCGATGCTCTTAGCTG and 1,516R-5′-CCCGGACATCTAAGGGCATC). In Figure 1, the amplification of PAFR in ALS mice and controls is shown. All three ALS-SOD1 mice (A1, A2, and A3) show higher PAFR expression as compared to controls (C1, C2, and C3). Gel densitometry analysis of the gel electrophoresis indicates that PAFR is in average 1.6 times overexpressed in ALS-SOD1 mice as compared to BL6 mice controls (Table 1).

**Table 1:** Densitometry of gel electrophoresis depicted in Figure 1.

Mouse	Area (pixels)	Mean	Min	Max
C1	330	75.003	31	140
C2	330	72.209	34	136
C3	330	71.718	36	122
A1	330	122.258	37	221
A2	330	108.527	39	251
A3	330	127.279	37	252

*Quantitation of specific gel band area by ImageJ software (https://imagej.nih.gov/ij/). A1, A2, and A3 are ALS/SOD1 mice and in C1, C2, and C3 the BL6 non ALS controls*.

## Discussion

Only two FDA approved drugs, Riluzole and Edaravone, are available currently for ALS therapy. Riluzole modulates glutamate neurotransmission by inhibiting both glutamate release and postsynaptic glutamate receptor signaling. Edaravone is a free radical scavenger (40). Riluzole increases patient survival by 3–6 months and relieves respiratory discomfort while the therapeutic effects of Edaravone are still controversial (41). A therapeutic possibility involving PAF antagonists *via* PAFR inhibition is discussed here. The rationale is that the augmented IL-18 in CSF in ALS patients is consistent with pilot experimental data indicating the overexpression of PAFR in SOD1 ALS mouse model. Several natural and synthetic PAF inhibitors are known, used with therapeutic purposes and can be tested for relief or cessation of ALS symptoms in the animal model.

A role for PFA and PAFR has been described in neuronal diseases such as Parkinson’s, epilepsy and stroke. The use of a PFAR antagonist in a human neuroblastoma cell line incubated with W7FW14F apomyoglobin amyloid aggregate increased cell viability when compared to control (42). PAF is increased after status epilepticus and the use of PAFR antagonist LAU-0901 reduces seizure susceptibility, restores and recoveries dendritic spine density, prevents dysmorphic filopodia-like projections and attenuates spontaneous epileptiform activities and dendritic spine changes in the hippocampus in pilocarpine mice model (43). A PAFR knockout mice model used in a transient global cerebral ischemia and reperfusion experiment showed that the lack of PAFR improves neurological deficits and decreases the percentage of necrotic cavities (44). PAF concentration was increased in perifocal regions of cerebral infarction after middle cerebral artery occlusion in rat and expression of PAFR was decreased following ischemia-reperfusion (45). In model of acute ischemic stroke on middle cerebral ischemia occlusion, rats pretreated with ginkgolide K had less infarction volume and oxidative stress index, such as superoxide dismutase and nitric oxide synthase were reversed to their normal levels in serum and in the cerebral ischemic section (46). Although PAF has already been study in other neuronal diseases, little is known about PAF’s effect on ALS.

## Future Directions

The upregulation of PAFR in CNS of ALS mice was tested in the lumbar section of the spinal cord. However, ALS affects glutamate synapses in the motor cortex. Lower motor neurons have acetylcholine synapses. Therefore, quantitative RT-PCR (47) should be performed not only in spinal cord RNA but also in motor cortex in the brain. More refined quantitation using quantitative PCR (48) should be performed to guarantee that the comparative expression is in the linear range of amplification reaction and not underestimated by signal saturation. Also, upregulation of PAFR peptide in the motor cortex and spinal cords should be tested with anti-PAFR antibodies to corroborate results obtained with mRNA upregulation.

The effect of PAFR inhibitors can be tested initially in ALS-SOD1 mice by oral administration of Ginkolide B with dosage appropriately scaled for mice. The effects on ALS symptoms, such as hind limb impairment can be tested against age matched controls.

If effective these compounds might prove a valuable tool in ALS therapy. The ALS mouse model used in pilot experiment is the SOD1-G93A gain of function model, therefore, it can be speculated that PAF inhibitors might have an effect at least for the treatment of the SOD1 ALS subtype which corresponds to 13% of FALS and about 1% of SALS.

## Ethics Statement

All animal experiments were performed in accordance with protocol #46763 approved by the IACUC of Pennsylvania State University.

## Author Contributions

MRSB conceived the hypothesis, planned performed the experiments and wrote the manuscript. AMS planned, performed theexperiments and edited the manuscript. EBN performed the experiments. JRC, JRB and RCF discussed the results and edited the manuscript.

## Conflict of Interest Statement

The authors declare that the research was conducted in the absence of any commercial or financial relationships that could be construed as a potential conflict of interest.
